# Sweet Side Streams: Sugar Beet Pulp as Source for High-Performance Supercapacitor Electrodes

**DOI:** 10.1021/acsomega.3c07976

**Published:** 2024-01-22

**Authors:** Julian Selinger, Kristoffer Meinander, Benjamin P. Wilson, Qamar Abbas, Michael Hummel, Stefan Spirk

**Affiliations:** † Institute of Bioproducts and Paper Technology, Graz University of Technology, Inffeldgasse 23, 8010 Graz, Austria; ‡ Department of Bioproducts and Biosystems, Aalto University, P.O. Box 16300, 00076 Aalto, Finland; § Department of Chemical and Metallurgical Engineering, Aalto University, P.O. Box 16200, 00076 Aalto, Finland; ∥ Institute for Chemistry and Technology of Materials, Graz University of Technology, Stremayrgasse 9, 8010 Graz, Austria

## Abstract

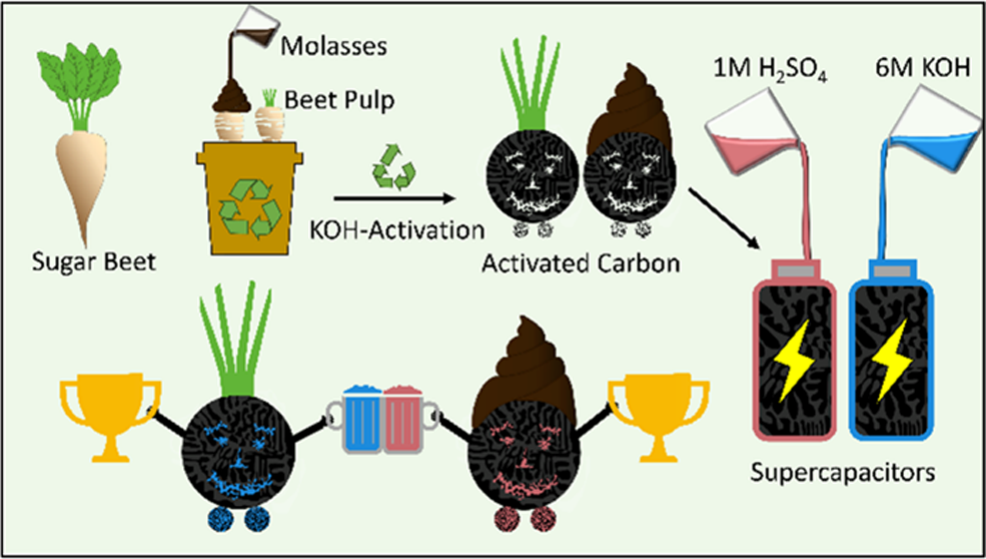

Valorization of the lignocellulosic side and waste streams is key to making industrial processes more efficient from both an economic and ecological perspective. Currently, the production of sugars from beets results in pulps in large quantities. However, there is a lack of promising opportunities for upcycling these materials despite their promising properties. Here, we investigate beet pulps from two different stages of the sugar manufacturing process as raw materials for supercapacitor electrodes. We demonstrate that these materials can be efficiently converted to activated, highly porous carbons. The carbons exhibit pore dimensions approaching the size of the desolvated K^+^ and SO_4_^2–^ ions with surface areas up to 2600 m^2^ g^–1^. These carbons were subsequently manufactured into electrodes, assembled in supercapacitors, and tested with environmentally friendly aqueous electrolytes (6 M KOH and 1 M H_2_SO_4_). Further analysis demonstrated the presence of capacitance-enhancing functionalities, and up to 193 and 177 F g^–1^ in H_2_SO_4_ and KOH, respectively, were achieved, which outperformed supercapacitors prepared from commercial YP80 F. Overall, our study suggests that side streams from sugar manufacturing offer a hidden potential for use in high-performance energy storage devices.

## Introduction

Beet processing generates large quantities of side streams, including molasses (ca. 4%) and lignocellulosic beet pulp (ca. 30%), which are currently either utilized as animal feed or subjected to incineration.^1^ The European Union (EU27) for instance is the world’s largest sugar beet producer with an annual harvest of 113 million tons.^2^ The industrial abundance of these beet pulp byproducts makes them promising starting materials for valorization to help foster a circular bioeconomy. Moreover, this issue has become more critical in recent years since the end of the EU sugar quota system in 2017 that led to a subsequent decline in the price of sucrose and prompted the sugar industry to search for additional sources of revenue.^3^ In addition, the readily available molasses and beet pulp side streams have the potential to be significant breakthroughs for the mitigation of climate change. Currently, upcycling efforts mainly focus on the utilization of beet pulp in biotechnology^4,5^ and material science applications.^6,7^ In addition, another promising route is the thermochemical conversion of beet pulp into activated carbon (AC) as such biobased materials have recently emerged as a valuable resource to create supercapacitor electrodes due to their environmental friendliness and low cost.^8^ One other major advantage of using biobased precursors for ACs is that they naturally contain heteroatoms, which may be incorporated into the carbon matrix through thermal treatment. The resulting heteroatom-doped functional groups do not significantly increase the mass load of the carbon structure because of their relatively low weight. The heteroatom-induced performance boost for supercapacitors is achieved through several combined effects, such as improved wettability, porosity, chemical active sites, charge density, and the implementation of redox-active groups.^9^

Supercapacitors are an attractive option for energy storage due to their high power density, fast charge and discharge rates, and long cycle life. They can effectively store and release energy quickly, making them useful in applications where rapid energy delivery is required (e.g., electric vehicles, portable electronics, etc.).^10^ Energy storage in SC relies on two main principles: physisorption and faradaic reactions. The physical energy storage mechanism depends on the electrolyte and pore size distribution of the AC. Meso- and macropores also play an important role in electrolyte transport, which can influence the overall power density.^10,11^ Micropores allow for a high number of ions to accumulate at the electrode surface and thus contribute disproportionately to the overall capacitance.^10,12^ The physical storage mechanism primarily occurs in pores below 10 nm,^13^ and when normalized to the surface area, the capacitance decreases with the pore size. Such behavior is valid up to the point when the hydrated ion and size of the pore reach parity as below the hydrated pore diameter, an anomalous increase in capacitance can be observed.^14,15^

Faradaic charge storage occurs via electrochemical redox reactions across the electrode/electrolyte interface^16^ and mesopores, predominantly above 10 nm, contribute disproportionately to this mechanism.^13^ Functionalization of the carbon structure can be achieved by incorporating heteroatoms (i.e., doping) that participate in Faradaic reactions. Typically, heteroatom doping can be achieved through post-treatments (e.g., plasma functionalization, chemical activation, microwave irradiation, etc.) or by following a self-doping strategy in which the precursor material consists of both carbon and the required heteroatom(s). Biomass is an excellent starting material for self-doping.^9^ This is by no means limited to one raw material but can be combined with biomasses of different compositions to achieve the desired final concentration of heteroatoms.

In this work, activated carbons from beet pulp are produced and incorporated into supercapacitors, along with well-known aqueous electrolytes. Two products at different steps from sugar manufacturing are compared: BP-UNM, which refers to beet pulp obtained after pressing but before drying, and BP-MOL, which refers to dried beet pulp impregnated with 10 wt % of molasses. Moreover, the utilization of thermochemical processing techniques leads to AC with a favorable pore size distribution, *i.e*., there is a high share of pores with diameters approaching the size of hydrated ions, while taking advantage of the heteroatom-rich material through the use of redox mediators.

## Materials and Methods

KOH (Merck, pellets), HCl (Merck, 37%), H_2_SO_4_ (VWR, 95%), acetone (VWR chemicals), and isopropanol (VWR, 98%) were purchased and used as received. Glass microfiber filters (GF/A from Whatman) were punched out (⌀ 10 mm) and used as separator in a Swagelok T-cell. LOCTITE EDAG PF 407C (Henkel, Düsseldorf, Germany) was used as a conductive adhesive to glue electrodes onto the stainless steel bolts. YP80 F from Kuraray and poly(tetrafluoroethylene) (PTFE) from Sigma-Aldrich were used.

### Precarbonization and Activation

Beet pulp was kindly supplied by the Agrana Research and Innovation Center (Tulln, Austria). Around 5 g of fully dry beet pulp (48 h at 105 °C) was placed in an Al_2_O_3_ crucible (7 × 4.5 × 1.5 cm^3^) and further transferred into a three-zone tube furnace (TZF 15/610, Carbolite, Neuhausen, Germany). By increasing the temperature (rate: 5 K min^–1^) to 400 °C and holding samples at the target temperature for 2 h in inert atmosphere (nitrogen flow: ca. 0.5 L min^–1^), precarbonized material (PCM) of beet pulp was obtained. Afterward, potassium hydroxide was added to the cooled PCM (PCM/KOH = 1:5; w-w) and this blend was then ground by means of pestle and mortar. Subsequently, the resultant pulverized mixture was heated using the same settings mentioned above, except that the target temperature was increased to 800 °C.

The obtained material was cooled to room temperature and afterward dispersed in hydrochloric acid (2 M, 120 mL). After stirring (600 rpm) for 1 h, the powder was washed with an excess of deionized water and filtered (0.8 μm mesh) in several repeating steps until the washing solution was pH-neutral. The resulting activated carbon (AC) was dried (24 h, 105 °C) prior to further processing.

### Scanning Electron Microscopy

Scanning electron microscopy (SEM) images were obtained by a Sigma VP (Carl Zeiss Microscopy, Jena, Germany) operated at a 0.7 kV acceleration voltage and detected with an in-lens detector. The raw materials (BP-MOL and BP-UNM) were coated with a 3 nm thick layer of iridium with an ACE600 sputter-coater (Leica Microsystems, Wetzlar, Germany). The ACs (AC_BP-MOL and AC_BP-UNM) were investigated without depositing a conductive layer.

### Transmission Electron Microscopy (TEM)

A sample grid (lacy carbon film on 300 mash copper grid) was placed in an evenly distributed AC:acetone dispersion (1:1000, w-w) for 1 s. Acetone was allowed to evaporate, and the grid was placed on a TEM sample holder. The sample was then investigated using a JEM-2800 (JEOL, Akishima, Tokyo, Japan) instrument with a 200 keV beam. Images were obtained via an ORIUS SC200 CCD camera (GATAN, Pleasanton, CA) with an exposure time of 0.6 s.

### Gas Adsorption Analysis

Gas adsorption measurements were performed with a Microtrac BELsorp Max II equipment (Microtrac Retsch GmbH, Haan, Germany) using nitrogen. Prior to measurement, individual samples were placed in separate glass measurement cells and subjected to an in situ heat pretreatment (300 °C for 24 h) under vacuum. Following successful pretreatment, multistep nitrogen gas adsorption measurements over a relative pressure range (*P*/*P*_0_) between 0 to 0.99 (0.99 to 0.3 for desorption) were undertaken in a liquid nitrogen bath at 77 K. Data was subsequently analyzed using BELMaster Analysis Software (Version 7.3.2.0, Osaka, Japan) to calculate the related multipoint Brunauer–Emmett–Teller (BET) sample surface areas and average pore size. In addition, pore size distributions were calculated from the adsorption branch of the appropriate gas isotherm using nonlocal density functional theory (NLDFT) based on a graphitic carbon adsorbent slit model with solid–fluid pore width definition and Tikhonov regularization fitting methodology.

### XPS Analysis

Measurements were performed with an AXIS Ultra DLD X-ray photoelectron spectrometer (Kratos, Manchester, U.K.) using a monochromated Al Kα X-ray source (1486.7 eV) run at 100 W. A pass energy of 80 eV and a step size of 1.0 eV were used for the survey spectra, while a pass energy of 20 eV and a step size of 0.1 eV were used for the high-resolution spectra. Photoelectrons were collected at a 90° takeoff angle under ultrahigh vacuum conditions, with a base pressure typically below 1 × 10^–9^ Torr. The diameter of the beam spot from the X-ray was 1 mm, and the area of analysis for these measurements was 300 × 700 μm^2^. Both survey and high-resolution spectra were collected from three different spots on each sample surface in order to check for homogeneity and surface charge effects.

Macrofibrillar cellulose in the form of an ash-free filter paper (Whatman, Maidstone, U.K.) was analyzed several times during the experiment as a reference to monitor possible contamination in the analysis chamber. All acquired spectra were charge-corrected relative to the position of the graphitic bonding of carbon at 284.2 eV.

In addition to the chemical analysis, a standard elemental analysis was undertaken with the goal of quantifying the surface functional groups through comparison of the O 1s spectra to previous work by Brender et al.^17^

### Elemental Analysis

A Thermo Scientific FlashSmart Elemental Analyzer (Waltham, MA) was used to measure the elemental composition (i.e., carbon (C), hydrogen (H), nitrogen (N), sulfur (S), and oxygen (O)) of the raw material as well as the respective ACs. Therefore, 1.5–3 mg of the sample was placed in a cup (tin for CHNS and silver for O measurements). For the AC investigation, 8–10 mg of vanadium pentoxide was added to act as a catalyst. As a standard for all measurements, BBOT (2,5-Bis (5-*tert*-butyl-benzoxazol-2-yl) thiophene) was used. The measurement was carried out under an inert (helium) and oxidative (oxygen) environment for O and CHNS determination, respectively.

### Raman Spectroscopy

The AC powder was pressed onto filter paper by means of a hydraulic press with a pressure of 2 kN cm^–2^. Afterward, three representative spots within the samples were investigated with an inVia Confocal Raman Microscope (Renishaw, Wotton-under-Edge, U.K.). A 532 nm laser (power 5%; 1 accumulation for 12 s) was shafted onto the sample, and the resultant signal was detected with a Centrus 05TJ55 (Renishaw, Wotton-under-Edge, U.K.). The spectra were fit with Lorentzian functions for the D- and G- bands, and their areal intensities were determined (*I*_D_ and *I*_G_, respectively). Subsequently, the crystallite size (*L*_a_) was calculated according to eq 1, being valid for a wavelength (λ_L_) between 400 and 700 nm.^18,19^
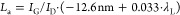
1

### Electrode Preparation and Supercapacitor Assembly

After manually grinding the AC for several minutes with a pestle and mortar, a slurry comprising AC, poly(tetrafluoroethylene) (PTFE) (Sigma-Aldrich St. Louis, MO), and Super P (Timcal, Bodio, Switzerland) was made by combining the separate components together. This was achieved by dispersion of 100 mg of AC, PTFE, and Super P (mixed in an 18:1:1 ratio) in 5 mL of isopropanol, which was placed on a magnetic stirrer (60 °C, 400 rpm) and agitated until the isopropanol evaporated (ca. 3 h). Subsequently, the slurry was placed on a clean glass surface and rolled between two aluminum spacers with a stainless steel cylinder to achieve an average thickness of 400 μm. After drying (105 °C, 24 h) the electrodes were punched (⌀ 6 mm), resulting in an average AC weight load of approximately 10 mg cm^–2^. In addition to the beet pulp-based electrodes, electrodes with YP80 F were also made for comparison with a commercial standard.

Further electrodes with the same weight (±0.1 mg) were paired and assembled in a Swagelok T-cell to an electric double-layer capacitor (EDLC). The two electrodes were each glued with EDAG PF 407C (Henkel, Düsseldorf, Germany) to a current collector (⌀ 10 mm stainless steel bolts) and separated with a GF/A glass fiber separator (⌀ 10 mm, Whatman, Maidstone, U.K.). Prior to tightening the system, 180 μL of electrolyte (1 M H_2_SO_4_ or 6 M KOH) was added.

### Electrochemical Characterization

All EDLCs were tested using a Biologic SP-150 potentiostat (Seyssinet-Pariset, France), and the cells were short-circuited for 15 min prior to measurement. For galvanostatic charge and discharge (GCD) and long-term measurements (LTM), the supercapacitor was charged and discharged between 0 and 0.8 V. For LTM, 7500 charge–discharge cycles were performed with a specific current of 2 A g^–1^ (based on the AC weight in one electrode), whereas GCD measurements were carried out at currents of 0.1, 0.3, 1, 2, 3, 5, and 10 A g^–1^, with 20 repetitions of each set. The floating test was conducted with five charge–discharge cycles between 0 and 0.8 V, followed by one charging cycle with a subsequent voltage hold at 0.8 V for 5 h. Those steps were repeated 22 times.

Cyclic voltammetry (CV) was undertaken in a measurement window between 0 and 0.8 V using scan rates of 2, 5, 10, 20, 50, and 100 mV s^–1^, respectively

Supercapacitors are usually used for applications where they are operated to half of their maximum voltage (0.5 *U*_max_). The capacitance (*C*) calculated from GCD data (also LTM and floating test) is based on the following assumption:^20^
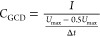
2where *I* is the applied current and Δ*t* the discharge time until half of the maximum voltage.

Capacitances from CV measurements were calculated based on eq 3:
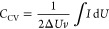
3where v is the scan rate and Δ*U* is the voltage range.^10,21^

The capacitances of *C*_GCD_ and *C*_CV_ (further denoted as *C*) reflect the capacitances for the whole cell. To calculate the specific capacitance for one electrode (*C*_sp,e_), a factor of 4 must be applied. The mass (*m*) is the active material (i.e., AC) in the cell.^20^
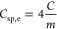
4

In this work, all values refer to the mass specific capacitance of one electrode (i.e., *C*_sp,e_), unless differently stated.

Equations 5 and 6 were used to calculate the energy density *E* and power density *P*, respectively, with Δ*t* being the discharging time.^10,21^
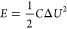
5
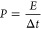
6

## Results

### Scanning Electron Microscopy and Transmission Electron Microscopy

**Figure 1:**
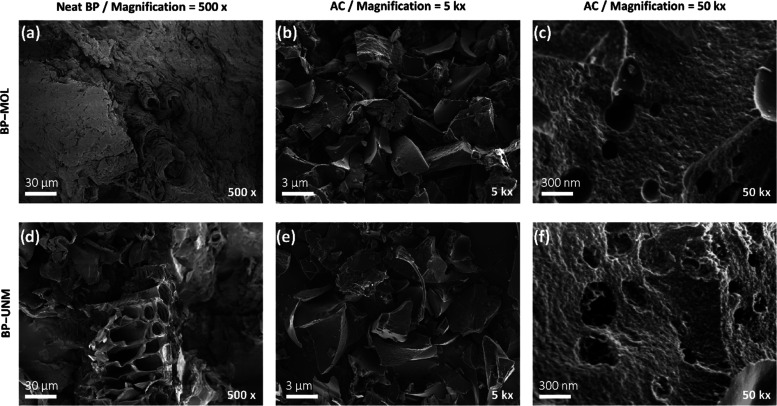
SEM images of BP-MOL (a), BP-UNM (d), and their corresponding ACs (AC_BP-MOL (b, c) and AC_BP-UNM (e, f)). The starting material exhibits a cellular structure. Both ACs show pores in the mesopore range.

The activated carbons were synthesized using a precarbonization step, followed by activation using solid KOH according to literature procedures.^22,23^ The characterization of the samples before and after carbonization is displayed in Figure 1. In both precursor materials (BP-MOL and BP-UNM), the cellular structure of beet pulp is clearly visible (Figure 1a,d). The ACs (AC_BP-MOL and AC_BP-UNM, Figure 1b,c,e,f) have a uniform appearance as porous grains. Macro- and mesopores (>50 and 2–50 nm, respectively, Figure 1c,f) can be observed in the higher-magnification SEM images. From both precursors, highly porous carbons were obtained whose morphological appearance is similar.

**Figure 2:**
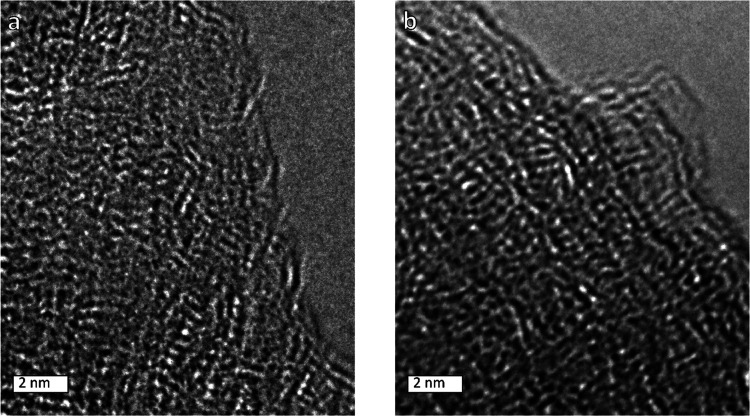
High-resolution TEM images of AC_BP-MOL (a) and AC_BP-UNM (b). Both materials comprise a disordered hierarchical structure with an interlayer distance of around 0.35 nm.

High-resolution transmission electron microscopy (HR-TEM) confirmed a disordered hierarchical structure for both samples (Figure 2). These results confirm the ultramicroporous nature and show a honeycomb-like structure and the graphitic nature of the material can be clearly observed in the images. The main part of the images consists of stacked graphitic layers, which have interlayer spacing channels of around 3.5 Å. This is consistent with literature data on HR-TEM of carbon materials.^24,25^

### Nitrogen Gas Adsorption Isotherms

**Figure 3:**
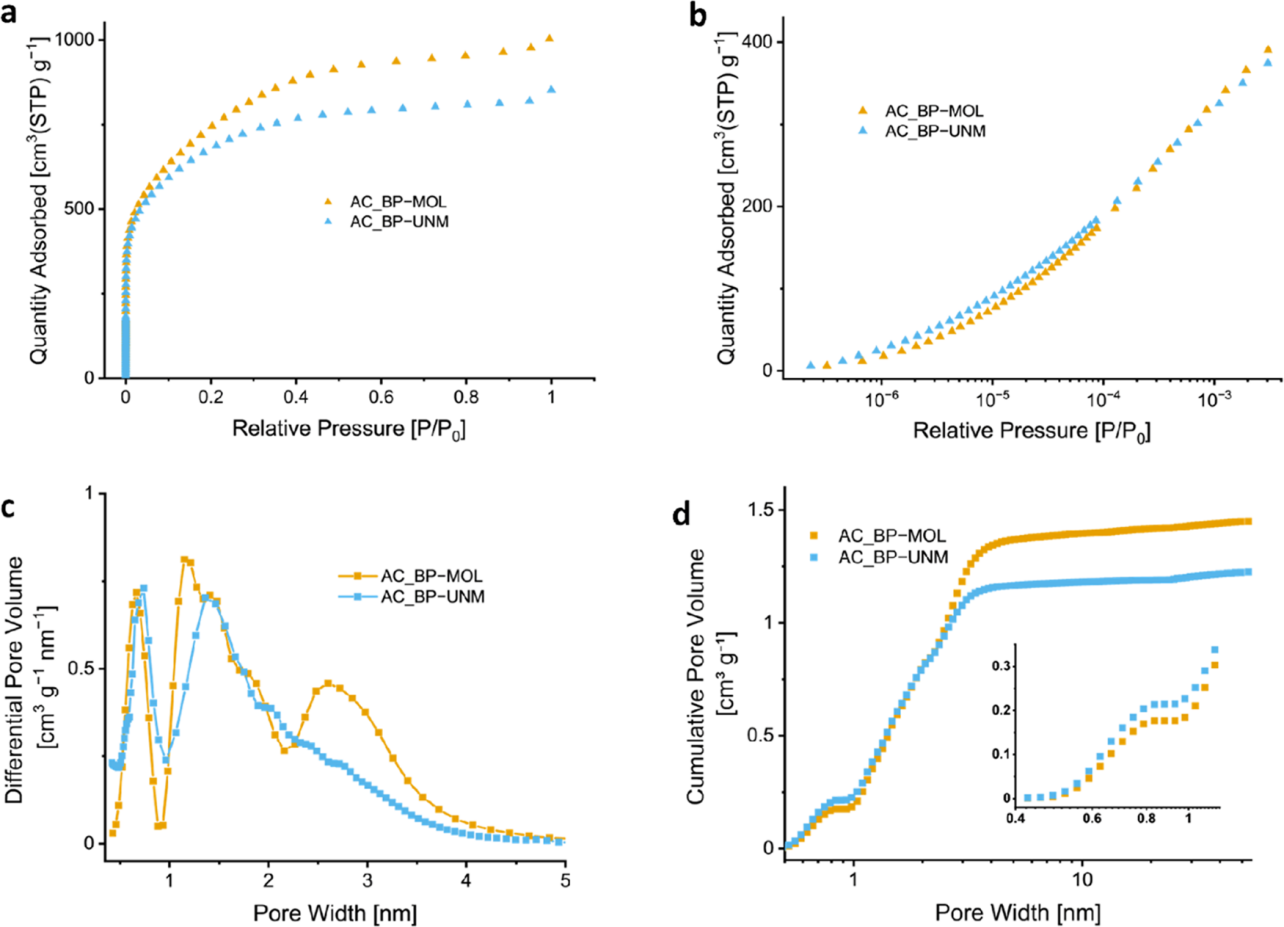
(a) Nitrogen adsorption isotherms with (b) highlighting the adsorption in the low-pressure regime on a logarithmic scale. (c) Pore size distribution for pores below 5 nm. (d) Cumulative pore volume in the micro- and mesoporous region, with the inset highlighting the pore width below 1.2 nm.

The isotherms presented in Figure 3a show the adsorption of nitrogen at 77 K over the entire pressure range (10^–8^ ≤ *P*/*P*_0_ ≤ 1). Figure 3b shows the low-pressure regime on a logarithmic scale, revealing the (ultra)microporous nature of the carbons. A rapid gas uptake at low relative pressure is associated with micropore filling. Both ACs display a type I(b) isotherm, indicating a broad range of pores, including wider micropores and narrow mesopores (<2.5 nm).^26^ These approximations are supported by the calculated pore size distribution diagram using an NLDFT model (Figure 3c). Both samples show a similar pore size distribution in the ultramicroporous regime (<2 nm), with AC_BP-UNM having a higher volumetric share (Figure 3d). The total pore volume for AC_BP-MOL is around 20% higher than that for AC_BP-UNM, exhibiting a clearly higher volumetric share of pores between 2 and 4 nm (Figure 3c). Consequently, the mean pore diameter is slightly smaller for AC_BP-UNM (1.08 nm) than for AC_BP-MOL (1.19 nm).^27^ The BET surface area was calculated to be 2600 and 2400 m^2^ g^–1^ for AC_BP-MOL and AC_BP-UNM, respectively.

As already demonstrated by TEM, the activated carbons contain a large share of pore widths below 0.8 nm. In general, pore channel widths below 10 nm are of high importance for the physical storage of the electrolyte.^13^ Pore channels approximating the size of the bare ion may lead to a large increase in capacitance, as demonstrated by the group of Gogotsi and co-workers.^14,15^ Considering the diameter of the hydrated/bare SO_4_^2–^ ion (7.6/5.8 Å) and the K^+^ ion (6.6/2.7 Å) a distortion of the solvation shell may occur.^28^ However, also sieving effects may occur, therefore, the emphasis should be placed primarily on the average pore size.^27^

### Raman Spectroscopy

**Figure 4:**
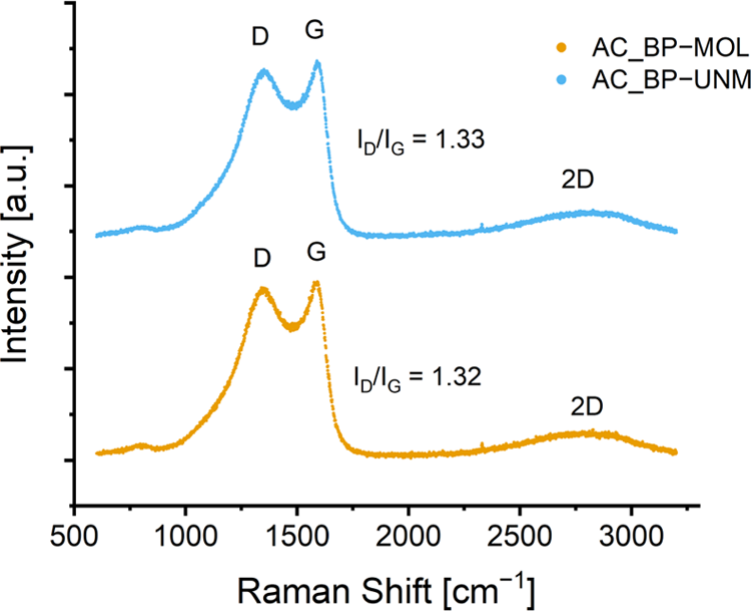
Raman spectra of AC_BP-MOL and AC_BP-UNM with the characteristic D, G, and 2D bands for carbon materials.

The spectra of both activated carbons (Figure 4) show the typical characteristics of turbostratic carbon, a type of disordered carbon material, with the D-band centered at around 1350 cm^–1^, the G-band at around 1590 cm^–1^, and the 2D band at approximately 2800 cm^–1^.^29^ From the fitted data (with Lorentzian functions), the *I*_D_/*I*_G_ ratio was calculated by integrating the curve to reflect the degree of graphitization. This resulted in similar values for both AC_BP-UNM and AC_BP-MOL (1.33 and 1.32, respectively), indicating no clear difference between the two materials. Using eq 1, the crystallite size *L*_a_ was calculated to be 3.7 nm.

### X-ray Photoelectron Spectroscopy

X-ray photoelectron spectroscopy (XPS) reveals the near-surface elements and allows for prediction of their functionalization. XPS shows similar elemental composition for both samples (C: 89.7 atom % vs 91.0 atom %, O: 8.0 atom % vs 7.4 atom %, N: 0.7 atom % vs 0.6 atom %, S: 0.7 atom % vs 0.5 atom % for AC_BP-MOL and AC_BP-UNM, respectively; the type of functional groups and their respective share are summarized in Table S2). Furthermore, XPS revealed a small amount of silicon present in the sample, stemming from the beet pulp samples (0.9 and 0.6 atom % for AC_BP-MOL and AC_BP-UNM*)*. The relatively low standard deviations (max 0.2 atom %) obtained from measurements at three different spots suggest that the samples are homogeneous. The C 1s spectra (Figure S2) were fitted with four Gaussian components according to standard tabulated chemical shifts, with peak positions at 284.8 eV (C–C), 286.5 eV (C–O), 287.8 eV (C=O), and 288.9 eV (O–C=O). The asymmetrical peak shape at 284.2 eV with two higher-binding-energy plasmons is typical of graphitic samples. Graphitic carbons account for around 75% in both samples for the highest share within the C 1s spectra.

**Figure 5:**
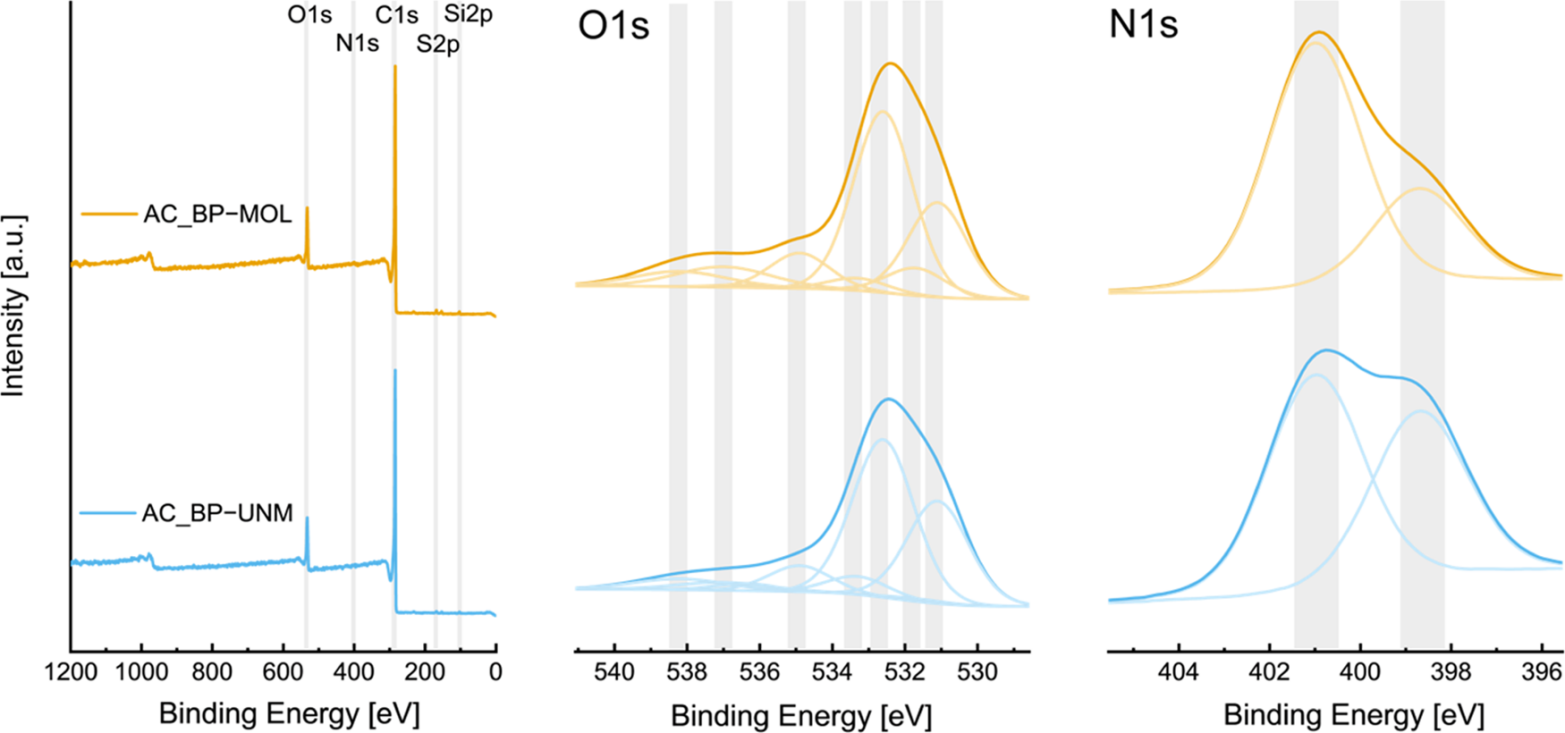
XPS spectra showing the full spectra (left) and high resolution of the O 1s deconvolution (middle) and the N 1s deconvolution (right) of AC_BP-MOL and AC_BP-UNM. Note that the intensities for the O 1s (8.0 atom % vs 7.4 atom %) and N 1s (0.7 atom % vs 0.6 atom %) are not scaled for better visibility.

A deconvolution of the O 1s spectra (Figure 5, middle) was performed using the same fitting parameters as those employed by Brender et al.^17^ The denoted source provides a rough estimation of the surface chemistry distribution. After deconvolution, the peaks indicate the presence of quinones (531.2 eV), carboxyls (531.8 eV, C=O), ethers (532.7 eV, C–O–C), and noncarbonyl oxygen in carboxyls or phenols (533.4 eV, C–O). Furthermore, water (535.0 eV), water in micropores (536.5 eV), satellites of carbonyls (537.1 eV), and satellites of carboxyls (538.3 eV) were detected. For all samples, the majority of oxygen was contained in varying degrees within quinones and ethers, indicating that oxygen content in the samples is not only caused by surface oxidation of the samples. The third most abundant form of oxygen is in phenols or carboxyls. AC_BP-MOL showed a relatively higher concentration of carbonyl oxygen in carboxyls.

The N 1s spectra were fitted with two Gaussian peaks at approximately 398.9 and 401.1 eV, which can be attributed to pyridine and pyrrole, respectively.^30^ Although the intensities from nitrogen were very low, the ratios for all spots within the sample are fairly similar. The sulfur S 2p spectra were fitted with S 2p_3/2_ peak at approximately 169.2, 168.0, 164.9, and 163.7 eV. The peaks at 168.0 and 169.1 eV can be probably assigned to inorganic sulfates while the peaks at 163.7 and 164.9 are probably due to organic aromatic sulfides/thiols.^31,32^

To summarize: the surface chemistry of the carbon comprises a wide range of functionalities ranging from carboxyls, phenols, and quinones groups. This impacts the properties of the supercapacitor, as the electrolyte may interact more efficiently with the carbon materials. For example, an alkaline electrolyte deprotonates the hydroxyl-ions of e.g., carboxylic groups.^33,34^ Carboxylic groups, however, may also limit the accessibility of the electrolyte, leading to worse cycling results.^9,35^

The presence of phenolic and quinone functional groups, which involve the transfer of up to two electrons during the reaction, is crucial for inducing Faradaic reactions.^36,37^ These functional groups enhance the wettability of the material and also contribute to its long-term capacitance.

Earlier studies suggest that the overall oxygen concentration impacts the supercapacitor’s performance more than its actual configuration.^38^ However, oxygen double bonds in aromatic or aliphatic carbon impact the conductivity to a higher degree. On the other side, singly bond oxygen to carbon (aromatic or aliphatic) has a positive effect on the capacitance.^38^

Nitrogen groups play a crucial role in improving supercapacitor performance.^39,40^ A nitrogen functionality within the carbon lattice is known to be more stable compared to embedded oxygen functional groups. Besides improving the wettability and electrochemically active sites, the presence of nitrogen-containing compounds contributes to pseudocapacitive effects (e.g., pyridine, pyrrole) in (aqueous) supercapacitors. Quaternary and pyridinic-*N*-oxides are known to improve the conductivity by a more efficient electron transfer.^9,13^ Because of the basic nature of N-5 and N-6, this effect is more pronounced in acidic media, as under alkaline conditions.^9^ The presence of sulfur groups may increase the wettability and conductivity and promote pseudocapacitive effects.^41,42^ The overall sulfur concentration is known to play a minor role in capacitance improvement, however, a high share of aromatic sulfide is primarily responsible for a sulfur-derived enhancement of capacitance.^43^

### Electrochemical Characterization

The ACs (AC_BP-MOL and AC_BP-UNM) and the commercial reference (YP80 F) were manufactured into free-standing electrodes (AC mass loading of 8–12 mg cm^–2^). The electrodes were further assembled as EDLC in a two-electrode configuration, and CV and GCD measurements were performed. The interaction between AC and the respective electrolyte (1 M H_2_SO_4_ or 6 M KOH) has been evaluated by means of capacitance, rate dependence, and cycling stability.

**Figure 6:**
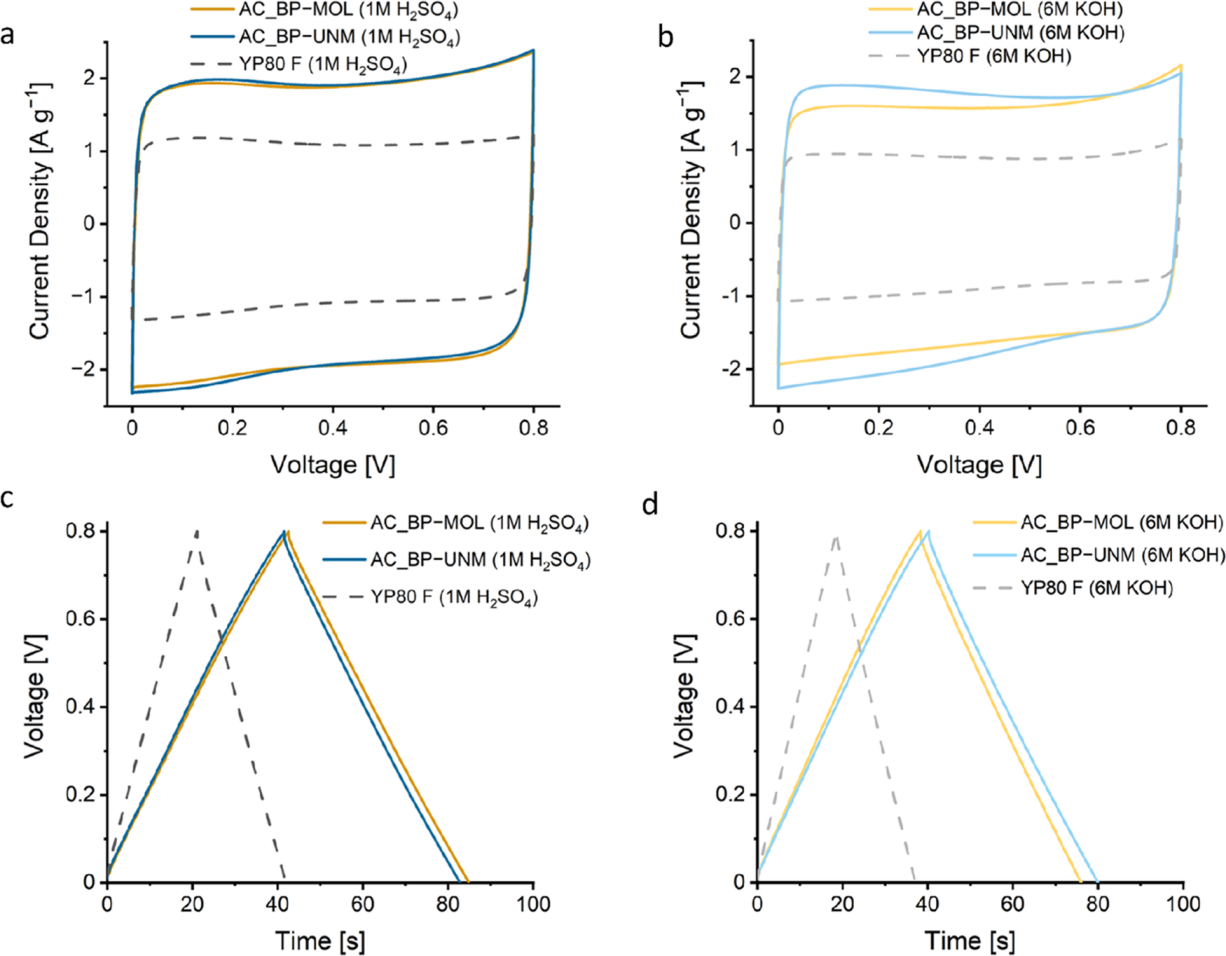
Cyclic voltammograms of the different supercapacitors at a cycling rate of 20 mV s^–1^ in 1 M H_2_SO_4_ (a) and 6 M KOH (b); GCD graphs at 2 A g^–1^ of the assembled supercapacitors with 1 M H_2_SO_4_ (c) and 6 M KOH (d). The dashed gray line indicates the reference material (YP80 F).

In theory, a supercapacitor with solely physisorptive charge storage is characterized by a rectangular CV shape.^44^ This definition is approximated by reference material YP80 F, regardless of the electrolyte used (Figure 6a,b). However, there is a minor Faradaic contribution evident in the CV curves, as indicated by deviations of the current density along the *x*-axis (Figure 6a,b). The beet pulp-derived ACs show a considerably higher proportion of the Faradaic contribution in the CV curves. This can be attributed to the presence of capacitance-enhancing functionalities, as indicated by XPS analysis.^44^

The GCD plots (Figure 6c,d) provide a similar picture, with an isosceles triangle suggesting a physisorptive storage mechanism. While the YP80 F shows almost no deviation from a linear slope, AC_BP-MOL and AC_BP-UNM deviate slightly. This suggests a minor contribution from redox reactions. The self-doping method proposed here results in a significantly lower pseudocapacitive contribution when compared to the heteroatom-induced approaches described in the literature.^13^ It should be noted that at all investigated current densities (0.1–10 A g^–1^), the beet pulp-derived SC electrodes outperform those assembled from YP80 F.

Regardless of the used electrolyte, both beet pulp-derived ACs clearly outperform the commercial reference YP80 F. With KOH as electrolyte, the capacitance (at 20 mV s^–1^) of AC_BP-UNM could even reach around 100% more than YP80 F (177 F g^–1^ vs 90 F g^–1^) in the used setup. Using H_2_SO_4_ as electrolyte, we reached a 72% higher capacitance (at 20 mV s^–1^), compared to YP80 F (193 F g^–1^ vs 112 F g^–1^, respectively). As for current density, the SC electrode from beet pulp exhibits at all investigated scan rates a larger capacitance than the commercial reference YP80 F. Comparing the in here tested carbons with state-of-the-art literature values derived from KOH-activated biomass (Table 1), it becomes evident that AC_BP-UNM and AC_BP-MOL perform comparably well. However, care should be taken in a direct comparison of those data because of slight variations in the testing setup as well as in the activation protocol.

**Table 1:** Supercapacitors from Biomass-Derived Potassium Hydroxide-Activated Carbons with KOH and H_2_SO_4_ as Electrolyte

biomass precursor	specific surface area [m^2^ g^–1^]	testing condition	electrolyte	specific capacitance [F g^–1^]	reference
Tasmanian blue gum	971	1 A·g^–1^	1 M KOH	212	(45)
seaweed	1493	0.5 A·g^–1^	1 M KOH	207	(46)
tea leaf	46	0.5 A·g^–1^	3 M KOH	132	(47)
coconut shell	2144	0.5 A·g^–1^	6 M KOH	98	(48)
walnut shell	1016	0.5 A·g^–1^	6 M KOH	169	(49)
sugar beet pulp	2400	20 mV s^–1^	6 M KOH	177	this work
Tasmanian blue gum	971	1 A·g^–1^	1 M H_2_SO_4_	110	(45)
teak leaf	486	1 mV s^–1^	1 M H_2_SO_4_	168	(50)
coffee silver skins	2500	2 A·g^–1^	1 M H_2_SO_4_	188	(51)
waste termites	1441	0.5 A·g^–1^	1 M H_2_SO_4_	92	(52)
sugar palm midrib	558	1 A·g^–1^	1 M H_2_SO_4_	210	(53)
molassed sugar beet pulp	2600	20 mV s^–1^	1 M H_2_SO_4_	193	this work

Comparing the performance of AC_BP-UNM and AC_BP-MOL in alkaline (KOH) and acidic (H_2_SO_4_) media, a clear influence on their maximum capacitance values becomes apparent. While the capacitance (at 20 mV s^–1^) of AC_BP-UNM (KOH) is 10% higher than that of AC_BP-DRY (KOH), the overall capacitance of both beet pulp SCs with H_2_SO_4_ is similar.

The reason for the better capacitance values of AC_BP-UNM(KOH) can be explained by its lower average pore diameter. Pores below 0.6 nm have been reported to have a positive impact on the areal capacitance with KOH, while above 0.6 nm, the areal capacitance in H_2_SO_4_ is increasing. This is attributed to the inaccessibility of the bigger SO_4_^2–^ ion to smaller pores.^54^ However, these effects are partly counteracted by the higher share of redox reactions occurring in acidic media (e.g., quinones). Thus, similar capacitance peaks (at 20 mV s^–1^) are reached with 1 M sulfuric acid (AC_BP-MOL: 192 F g^–1^; AC-BP-UNM: 193 F g^–1^).

**Figure 7:**
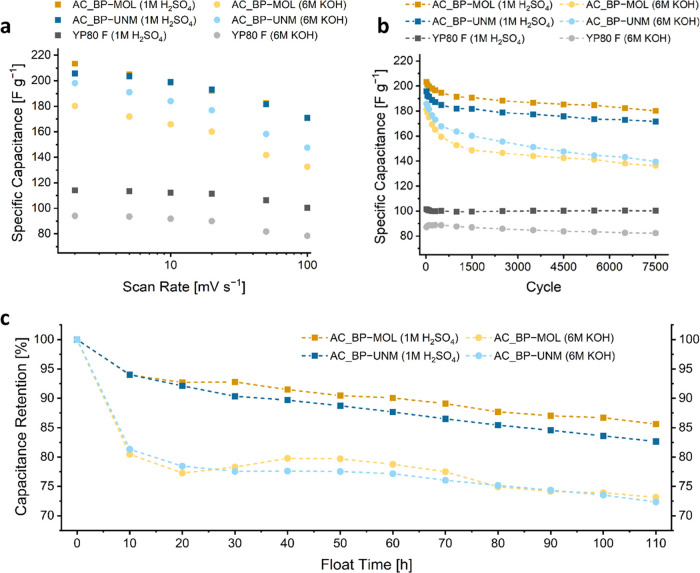
Specific capacitance of AC_BP-MOL, AC_BP-UNM, and YP80 F tested with 1 M H_2_SO_4_ and 6 M KOH electrolytes as a function of (a) the scan rate of CV measurements and (b) cycle number during long-term cycling at 2 A g^–1^. (c) Capacitance retention of AC_BP-MOL and AC_BP-UNM with 1 M H_2_SO_4_ and 6 M KOH as electrolytes during the floating test over 110 h holding at 0.8 V, with charge–discharge rates of 0.2 A g^–1^.

Regardless of the electrolyte, both beet pulp-derived ACs show a similar scan rate dependence (Figure 7a). For KOH, both ACs show a retention of around 74% from 2 to 100 mV s^–1^, while YP80 F retains 83% capacitance. SCs assembled with H_2_SO_4_ retain 80, 83, and 88% for AC_BP-MOL, AC_BP-UNM, and YP80 F, respectively. In general, the electrolyte has a higher influence on the capacitance than the carbon material used carbon material. One of the reasons might be the presence of sulfur groups in the beet pulp-derived AC, which are known to be unstable under KOH at higher rates.^9^

Frackowiak and Beguin reported that KOH is known to hinder pseudocapacitive effects over a longer range of cycles.^44^ We could observe this behavior for long-term measurements (Figure 7b). The capacitance retention after 7500 cycles with KOH is 75% while for H_2_SO_4_ it is 88%, independent of the used beet pulp precursor. However, it should be mentioned that AC_BP-UNM(KOH) shows a steeper slope with an advancing cycle number. The degradation of unstable functionalities (e.g., carboxyls, phenols) may first lead to a reduced redox activity and second reduce the accessibility to smaller pores. The effect of this is that physisorptive charge storage is hindered.^9^ This might be also the reason for a similar trend shown by floating tests (Figure 7c), which simulate a more realistic and demanding scenario as repeating cycles.^55^ The capacitance decay after 10 h of floating was 6% and around 19% for H_2_SO_4_ and KOH electrolyte, respectively. With consecutive floating time AC_BP-MOL(H_2_SO_4_) is more stable with a capacitance retention of 86% after 110 h, compared to AC_BP-UNM(H_2_SO_4_) with 83%. However, KOH seems to have a similar impact on both carbons’ performance, with a capacitance retention of around 73% for AC_BP-MOL(KOH) and AC_BP-UNM(KOH).

Although the capacitance retention after floating and cycling is rather low, it should be highlighted that after 7500 cycles, the performance of the supercapacitors equipped with the beet pulp-derived carbons still outperforms the commercial reference by 40–80% in the used setup.

## Conclusions

In search of new ways to generate added value from byproducts of sugar beet processing, we took a close look at the sugar production line. Neat beet pulp and beet pulp with the addition of molasses were used as a precursor to produce activated carbons. The impact of the precursor material on the pore size distribution and surface area of the activated carbon were evaluated. Finally, their suitability as supercapacitor electrodes was investigated through the assembly of different supercapacitor cells that included two distinct electrolytes (1 M H_2_SO_4_ and 6 M KOH).

We demonstrated that side streams from sugar manufacturing are suitable precursors for supercapacitors. Surface areas up to 2600 m^2^ g^–1^ (AC_BP-MOL) could be achieved, with pores down to the size of the electrolyte naked ion. The starting material had a decisive influence on the pore distribution and surface functionalization. With the addition of molasses to the beet pulp precursor the average pore diameter, as well as the surface area increased. The addition of molasses to the beet pulp also had an impact on the functionalization of the activated carbons.

However, the addition of molasses does not necessarily contribute significantly to the overall capacitance. Even though the influence on redox-active functionalities turned out to be positive, the total impact in terms of cycling stability and overall performance is rather low. Furthermore, the areal capacitance is lower than that for the neat beet pulp carbons (AC_BP-MOL). The neat beet pulp-derived carbons could outperform the commercial reference (YP80 F) by 97% with 193 and 177 F g^–1^ (at 20 mV s^–1^) independent of the electrolyte used.

Although there is a rather significant capacitance fading as a function of cycles, the capacitance of the beet pulp materials still outperforms that of the YP80 F reference material by 70% and more.

Overall, our findings offer valuable insight into the potential of currently underutilized side streams from industrial sugar manufacturing as renewable resources for energy applications. The results underscore the importance of ongoing innovation in this field, which can aid in the transition toward a more sustainable and circular economy.

## Supporting Information Available

XPS spectra of C 1s and S 2p deconvolution, CV at 2 mV s^–1^ and GCD measurements at 0.1 A g^–1^, Ragone plot of the carbons tested, elemental composition of AC_BP-MOL and AC_BP-UNM, and detailed XPS evaluation and all capacitances calculated based on CV and GCD measurements (PDF)
