# Supplementary material for: Sweet Side Streams: Sugar Beet Pulp as Source for High-Performance Supercapacitor Electrodes
Source: ACS Omega. 2024 Jan 22;9(4):4733–43. doi: 10.1021/acsomega.3c07976 (PMC10831825; doi:10.1021/acsomega.3c07976)
Supplement: Supplementary file 1 — ao3c07976_si_001.pdf [file ao3c07976_si_001.pdf]

Sweet Side Streams: Sugar Beet Pulp as Source for High-Performance Supercapacitor Electrodes

Julian Selinger[†,‡], Kristoffer Meinander[‡], Benjamin P. Wilson[§], Qamar Abbas[||], Michael Hummel[‡] and Stefan Spirk[†,]*

† Institute of Bioproducts and Paper Technology, Graz University of Technology, Inffeldgasse 23, 8010 Graz, Austria.

‡ Department of Bioproducts and Biosystems, Aalto University, P.O. Box 16300, 00076 Aalto, Finland.

§ Department of Chemical and Metallurgical Engineering, Aalto University, P.O. Box 16200, 00076 Aalto, Finland.

|| Institute for Chemistry and Technology of Materials, Graz University of Technology, Stremayrgasse 9, 8010 Graz, Austria.

*stefan.spirk@tugraz.at

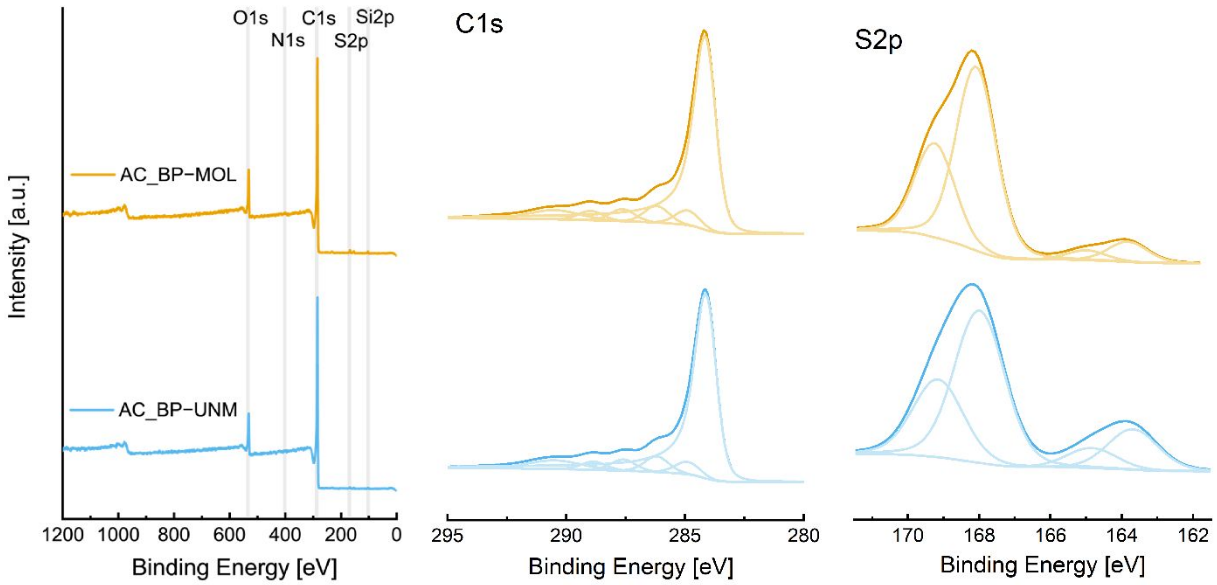

Figure S1. XPS−spectra showing the full spectra (left), and high resolution of the C1s deconvolution (center) and the S2p deconvolution (right) of AC_BP−MOL and AC_BP−UNM. Note that the intensities for C1s (89.7.0 vs 91.0 at%) and S2p (0.7 vs 0.5 at%) are not scaled for better visibility.

Figure S2. Cyclic voltammograms of the different supercapacitors at a cycling rate of 2 mV s^{-1} in

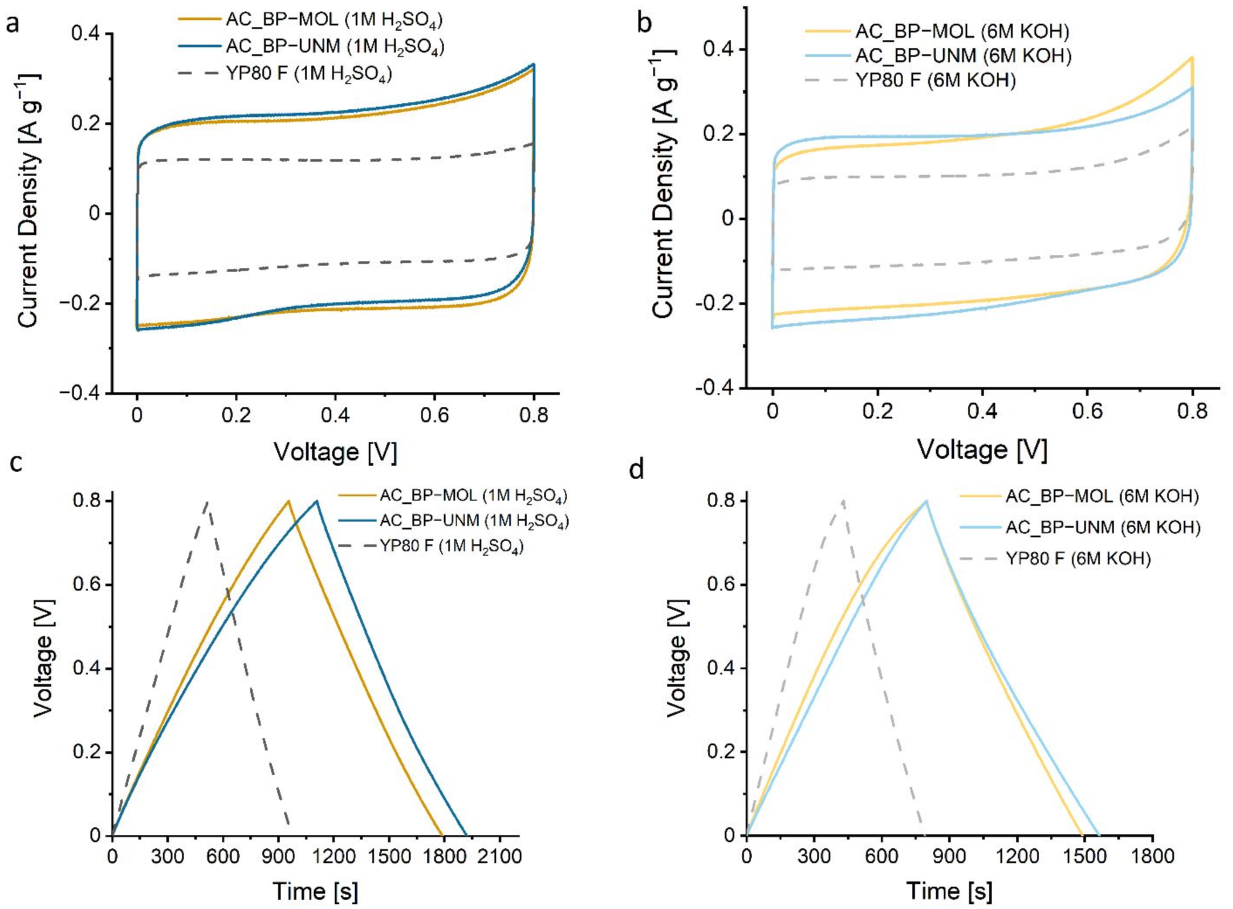

1M H_2SO_4 (a) and 6M KOH (b); GCD graphs at 0.1 A g^{-1} of the assembled supercapacitors with 1M H_2SO_4 (c) and 6M KOH (d). The dashed grey line indicates the refrence material (YP80 F).

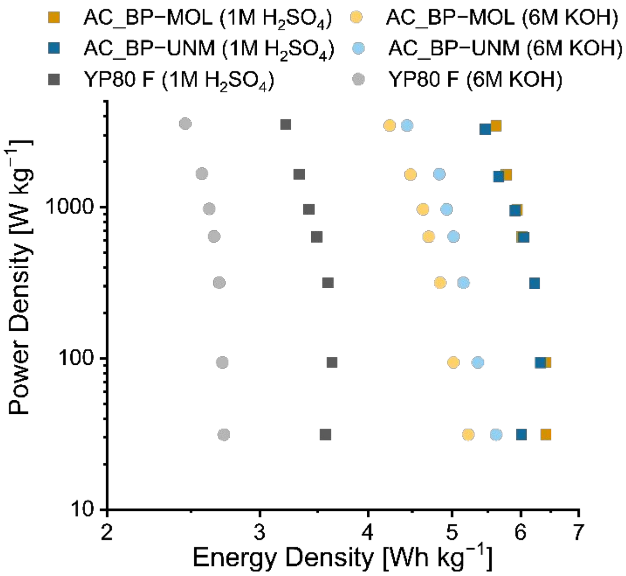

Figure S3. Ragone Plot from different of AC_BP−MOL, AC_BP−UNM and YP80 F tested with 1M H_2SO_4 and 6M KOH as electrolyte.

Table S1. Elemental composition of the precursor material and the activated carbons.

	**Elemental Analysis – bulk**				**XPS – surface**	
	Precursor		Activated Carbon		Activated Carbon	
Element	BP−MOL [%]	BP−UNM [%]	BP−MOL [%]	BP−UNM [%]	BP−MOL [%]	BP−UNM [%]
Carbon	43.1 (± 0.4)	44.7 (± 0.2)	88.6 (± 0.1)	88.2 (± 0.1)	89.7 (± 0.1)	91.0 (± 0.2)
Oxygen	—	—	5.1 (± 0.1)	5.7 (± 0.0)	8 (± 0.1)	7.4 (± 0.2)
Hydrogen	5.8 (± 0.1)	6.1 (± 0.1)	1.1 (± 0.0)	0.9 (± 0.0)	—	—
Nitrogen	1.8 (± 0.1)	1.7 (± 0.0)	0.6 (± 0.1)	0.7 (± 0.0)	0.7 (± 0.2)	0.6 (± 0.0)
Sulfur	0.2 (± 0.0)	0.2 (± 0.0)	0.8 (± 0.0)	0.8 (± 0.0)	0.7 (± 0.0)	0.5 (± 0.0)
Silicon	—	—	—	—	0.9 (± 0.1)	0.6 (± 0.0)
Ash	7.1 (± 0.1)	4.2 (± 0.1)	—	—	—	—

Table S2. XPS Analysis of the activated carbons AC_BP−MOL and AC_BP−UNM. The values are given as relative share of the to the whole spectra (left column) and the assigned element (right column).

	Relative share to the whole spectra [%]		Relative share to the assigned element [%]	
	AC_BP−MOL	AC_BP−UNM	AC_BP−MOL	AC_BP−UNM
**OXYGEN O1s**				
quinones	1.9	2.3	23.5	30.6
Carbonyl oxygen in carboxyls	0.6	0.1	7.3	0.7
ether	3.6	3.4	44.6	46.2
Noncarbonyl oxygen in carboxyls. phenols	0.2	0.4	2.2	5.1
water	0.7	0.6	8.7	7.7
Water in micropores	0.1	0.1	1.0	1.8
Satellite of carbonyls	0.5	0.3	6.2	4.0
Satellite of carboxyles	0.5	0.3	6.6	4.0
	AC_BP−MOL	AC_BP−UNM	AC_BP−MOL	AC_BP−UNM
**CARBON C1s**				
graphite %	74.3	76.9	82.9	84.5
C-C %	4.5	3.5	5.0	3.9
C-O %	4.7	4.7	5.3	5.1

C=O %	3.5	3.4	3.9	3.8
O-C=O %	2.6	2.5	2.9	2.8
	AC_BP–MOL	AC_BP–UNM	AC_BP–MOL	AC_BP–UNM
**NITROGEN N1s**				
pyrrole	0.5	0.3	72.4	56.5
pyridine	0.2	0.2	27.6	43.5
	AC_BP–MOL	AC_BP–UNM	AC_BP–MOL	AC_BP–UNM
**SULPHUR S2p**				
169.2	0.2	0.2	35.6	33.4
168	0.4	0.3	64.4	66.6

Table S3. Capacitances at different current densities.

Current Density [A g−1]	YP80 F (H_2SO_4) [F g^{-1}]	YP80 F (KOH) [F g^{-1}]	AC_BP−MOL (H_2SO_4) [F g^{-1}]	AC_BP−MOL (KOH) [F g^{-1}]	AC_BP−UNM (H_2SO_4) [F g^{-1}]	AC_BP−UNM (KOH) [F g^{-1}]
0.1	102.93	78.61	184.83	150.33	173.12	161.88
0.3	104.71	78.26	184.51	144.54	182.14	154.25
1.0	103.55	77.60	179.29	139.57	179.28	148.47
2.0	100.51	76.54	173.33	135.37	174.24	144.57
3.0	98.43	75.60	171.21	133.36	170.28	141.94
5.0	95.95	74.12	166.33	129.00	162.99	139.20
10.0	92.62	70.92	161.93	122.06	157.25	127.79

Table S4. Capacitances at different scan rates.

Scan Rate [mV s^{-1}]	YP80 F (H_2SO_4) [F g^{-1}]	YP80 F (KOH) [F g^{-1}]	AC_BP−MOL (H_2SO_4) [F g^{-1}]	AC_BP−MOL (KOH) [F g^{-1}]	AC_BP−UNM (H_2SO_4) [F g^{-1}]	AC_BP−UNM (KOH) [F g^{-1}]
2	114.02	94.02	213.43	180.23	205.67	198.17

5	113.46	93.52	205.19	171.88	203.61	191.02
10	112.25	91.79	198.80	165.82	198.99	184.02
20	111.54	89.95	192.18	160.10	193.09	176.83
50	106.37	81.70	182.30	141.72	181.50	158.22
100	100.42	78.39	170.69	132.62	170.94	147.58
